# The Royal Army Medical Corps: How Matters Stand To-Day

**Published:** 1915-09-04

**Authors:** 


### THE ROYAL ARMY MEDICAL CORPS.

### How Matters Stand To-day.

A few years ago far-reaching alterations in status and in pay were brought about in the Royal Army Medical Corps (R.A.M.C.), which were, in the main, very decided improvements, and had the effect of rendering this service very much more attractive than it had formerly been. To these changes and the resulting raised standard of attainments and efficiency among the officers recruited in recent years is partly—indeed, very largely—due the fine record which the R.A.M.C. has established for itself in the present war. Hitherto admission to the Royal Army Medical Corps has been obtainable by a competitive entrance examination, held twice a year; but just at present this examination is, wisely, in abeyance, and admission to the permanent establishment of the Corps is by nomination—practically always, that is, through the medium of either the Special Reserve, the Territorial Force, or the long list of temporarily commissioned officers. No doubt when peace is declared, or within a reasonable period thereafter, a return will be made to the examination system under regulations more or less similar to those in force before the war. Anyone anxious to know the system by which officers of the Regular R.A.M.C. qualified for their commissions before the war is referred to our exhaustive article on this subject last year (The Hospital, September 5, 1914, pp. 623 *et seq.*).

It is provided in the regulations that on admission to the Corps by examination the rank of lieutenant (on probation) is granted, with the pay of that rank. This commission has to be confirmed, which is done if the young officer successfully passes certain qualifying examinations held after he has gone through a definite course of instruction in recruiting duties, in military surgery, in tropical medicine, in hygiene, in pathology, in medical administration, in regimental duties, and in drill and field training. To allow of this being carried out satisfactorily, he is required to attend, immediately on his appointment as temporary lieutenant, courses of instruction at the Royal Army Medical College, Millbank, London, and at the Royal Army Medical Corps School of Instruction at Aldershot. Whether, in the special circumstances of the moment, this course of instruction is carried on exactly as in peace time the Regulations do not say: probably it has been modified to meet the pressing requirements of the moment, or even omitted altogether in favour of practical training in the field.

The physical, as opposed to the scientific and professional, examination of the candidates is doubtless as stringent as in peace time. This physical examination is not a severe one, but is in every way a thorough and searching one. In it attention is directed to the correlation of age, height and chest measurement, and to the weight. A good deal of importance, too, is attached to the power of vision. The Army Test Types and Snellen's "Optotypi" (Editiòn 1902) are used for determining the visual acuteness. If a candidate can read D6 at 6 metres (20 English feet) and D0.6 at any distance selected by himself, with each eye without glasses, he will be considered "Fit." At the same time a moderate degree of myopia is not considered a disqualification, provided that it can be corrected by glasses so as to secure adequate vision for the performance of operations, and that no organic disease exists. The other matters brought under observation in this physical examination are the hearing, absence of stuttering, the condition of the teeth (loss or decay of the teeth being considered disqualification), the state of the chest organs, the existence of rupture, of varicocele, of any chronic skin disease or any congenital malformation or defect, and of any evidence of previous acute or chronic disease.

Hitherto—that is to say, up to the outbreak of war in August 1914—various concessions were made by the military authorities to candidates who at the date of passing the entrance examination held, or were about to hold, a resident appointment in a recognised civil hospital. Should the candidate desire to fulfil the engagement he could be seconded for a period of a year to allow him to hold the appointment, and it was arranged that his future prospects in the Service should not suffer, except in one respect—namely, that he would receive no pay from Army funds during the time he held his civil appointment.

It is probable that just now this arrangement also is in abeyance: for one thing, it is difficult to conceive of any young medical man wishing to adopt the Army as his profession and not burning to get abroad at the earliest possible moment. The difficulty of the hospitals in getting young men for their resident appointments is clear enough evidence of this, for even those young men who take temporary commissions only, and have no intention of remaining in military employ after the war, are refraining from doing resident appointments in order to join the Army at once. Only a week ago we published (see The Hospital, August 28, 1915, p. 460) details of a new scheme by which the resident staffs of hospitals with medical schools are granted honorary commissions in the R.A.M.C. at all hospitals connected with medical schools, conditional upon their being available for general service with the R.A.M.C. after three months' residence at their hospital. The effect of this scheme will be that the teaching hospitals will be to some extent secured against losing resident medical officers, and also that young medical men going to the Front will have some special training before they go. The mere fact, however, of such an arrangement being necessary in the interests of the hospitals is a clear enough indication of the difficulties which the war is imposing on them; and it is a legitimate deduction that young doctors taking permanent commissions in the R.A.M.C. are not very likely to be applying for that permission to second for a year into civil employment which the regulations allowed them to do before the war.

### The Meaning of "Pay."

What, then, are the prospects, immediate and remote, of the R.A.M.C. as a career? When speaking of the pay of an Army medical officer, it must be remembered that in addition to the actual daily money he receives he is provided by Government with a servant, with lodgings, and with fuel and light, so that all these latter must be taken into consideration in reckoning his annual emolument. Thus, the actual monetary pay of a lieutenant on joining is £255 10s., but with the allowances mentioned above it amounts to £326 annually, it being, however, understood that if Government provides accommodation in barracks and fuel and light the allowances for these are not given, so that under these circumstances the lieutenant will only get his £255 10s. plus an allowance of £18 5s. for a servant, which gives him £273 15s. per annum. In addition to this, there is, when on active service abroad, "field allowance" to be added; this additional allowance varies according to rank from half-a-crown a day upwards. Bearing this explanation in mind, the following are the rates of pay and allowances for Army medical officers when serving at home, a different scale coming into force, as we shall see, when they do duty abroad.

| | Pay. | | | With Allowances, but exclusive of Field Allowance. | | |
|---|---|---|---|---|---|---|
| | £ | s. | d. | £ | . | . |
| Lieutenant ... ... ... | 255 | 10 | 0 | 326 | 0 | 0 |
| Captain... ... ... ... | 282 | 17 | 6 | 372 | 1 | 9 |
| Captain, after 7 years' full-pay service ... ... ... | 310 | 5 | 0 | 399 | 9 | 3 |
| Captain, after 10 years' full-pay service ... ... ... | 383 | 5 | 0 | 472 | 9 | 3 |
| Major ... ... ... ... | 428 | 17 | 6 | 583 | 9 | 7 |
| Major, after 3 years' service as such ... ... ... | 474 | 10 | 0 | 629 | 2 | 1 |
| Lieut.-Colonel ... ... ... | 547 | 10 | 0 | 711 | 4 | 7 |
| Lieut.-Colonel, after 3 years' service as such ... ... | 638 | 15 | 0 | 802 | 9 | 7 |
| Colonel ... ... ... ... | 821 | 5 | 0 | 1,008 | 16 | 1 |
| Surgeon-General ... ... | 1,095 | 0 | 0 | 1,447 | 16 | 3 |

Under certain conditions additional pay is given, as when a medical officer is in charge of a general or other hospital, and the higher staff appointments in the R.A.M.C. have their own special remuneration; the Director-General, for instance, receiving £2,000 per annum, while officers appointed professors and assistant-professors at the Royal Army Medical College receive the pay and allowances of their rank, plus £200 and £80 per annum respectively.

This additional pay is in normal times drawn by comparatively few officers, usually of the rank of lieutenant-colonel or major. But in war the establishments are so vastly expanded that at the present time a high proportion of the field officers of the R.A.M.C. are in receipt of augmented pay, and quite a number of junior officers are in the same fortunate case. Thus the officer commanding a field ambulance or any other medical unit (such as a casualty clearing station or a general or stationary hospital) is entitled to command pay. This is usually ten shillings a day, or £182 10s. a year. Normally such an officer would be a lieutenant-colonel, but nowadays it is not at all uncommon for majors to hold these posts, and to receive the handsome addition to their pay represented by command pay, which is, of course, irrespective and independent of the rank of the recipient. Even captains have been known to command medical units, and there would seem to be nothing in the regulations to prevent them receiving command pay.

Another source of additional pay now open to junior officers of the R.A.M.C. is the half-crown a day payable to any officer under the rank of lieutenant-colonel holding an appointment as specialist. In a general hospital, for instance, there are two such paid specialists, a surgeon and a bacteriologist respectively; *x*-ray specialists are apparently not entitled to this additional pay, at any rate in general and stationary hospitals on active service. This specialist pay goes, as a rule, to a captain or a lieutenant—frequently to an officer holding a temporary commission. Another source of additional income for officers of the R.A.M.C. is charge pay. It is laid down that an officer in charge of a general or other hospital, or of a division of a general hospital, shall be paid, if in charge of at least 50 beds, half-a-crown daily; if of at least 100 beds, 5s.; of at least 200 beds, 7s. 6d.; of at least 300 beds, 10s. In peace time such appointments are few in number and much sought after; as a rule they are allotted to senior officers. But in war-time they become much more numerous, and are sometimes filled by quite junior officers. There have been in this war instances of lieutenants having charge of divisions of hospitals containing 300 beds, and actually receiving the 10s. a day charge pay for this duty. Naturally, since no medical officer could himself attend to 300 wounded men, such charges involve the command of other officers and also the administrative work connected with a large hospital and its staff; so the pay is not out of proportion to the responsibilities of which it is the recognition. Still, it is a good instance of the opportunities of increased pay and credit which war brings to the junior as well as to the senior officers of the R.A.M.C.

### Service Abroad.

In ordinary times the Army medical officer is liable to be sent abroad, the number required for this foreign service varying from time to time. As a rule a lieutenant is sent abroad during the second year of his service, the greater majority of them going to India. The term of service abroad is five years for India, the Mediterranean, and South Africa, and three years at other stations. When an officer's turn arrives for foreign service he is duly warned some months before he is required to embark, and has the opportunity of naming his preference for any particular station abroad, his wishes being met as far as Service exigencies permit. When stationed abroad an Army medical officer receives a higher scale of pay. Thus, in

India a lieutenant receives 420 rupees per month, which represents annual pay of about £500 if the rupee is reckoned at its supposed value of two shillings. Other ranks show a corresponding increase, and in view of the fact that service in India in the present day is passed under much more favourable climatic and hygienic conditions than formerly prevailed, by many it is preferred to service at home and has many advantages. This is seen in the very keen competition which prevails for what is known as "The Indian Medical Service," which is quite distinct from the R.A.M.C., the members of it undertaking to serve continuously in India with what is called "The Indian Army," made up of both British and native troops. The qualifying conditions and examinations (compare the article on page 489) are very much the same as for the R.A.M.C., but the officers on appointment are at once sent to India by troopship or otherwise at the public cost, and they spend all their service abroad. Naturally the pay and allowances, the leave and rules, the invalid and retiring pensions, and the half-pay are different from those of the home service, and are, speaking generally, on a higher scale.

Another fact that should be mentioned is that officers of the R.A.M.C. are permitted to accept employment under the Foreign or Colonial Offices when the interests of the public service make it desirable. By this arrangement they may be employed with the Medical Service of the Egyptian Army, with the Sanitary Service of the Egyptian Government, or with the Medical Services of any foreign or Colonial Government. While in this position they are specially paid by the Government retaining them, but their service counts towards promotion and possibly pension in the R.A.M.C.

### Promotion.

In the matter of promotion in the R.A.M.C., an officer is eligible to pass to the rank of captain on the completion of three and a half years' service and to the rank of major on the completion of twelve years' total service, provided that in each case he has passed the necessary examination and is recommended for such promotion. The examination for captain's rank may be taken at any time after completing twelve months' service, and is confined in its subjects to practical tests in map reading and problems in connection with practical tactics, to military law, to organisation, administration, and equipment, and to military medical administration, the duties of executive medical officers, and the terms of the Geneva Convention. The major's examination may be taken after five years' service, and includes the subjects above mentioned, along with medicine, surgery, hygiene, bacteriology, and tropical diseases, together with one special subject, such as bacteriology, dermatology, advanced operative surgery, ophthalmology, otology, and several others. A knowledge, too, is required of R.A.M.C. drills and exercises. Above the rank of major promotion is not by any length of service, but by selection, and is dependent on vacancies. A major, however, before he can be promoted to lieutenant-colonel must pass a qualifying examination. This can be taken at any time after three years in the rank of major and is in the following subjects:—

1. Army medical organisation in peace and war.
2. Sanitation of towns, camps, transports, and all places likely to be occupied by troops in peace and war; epidemiology, and management of epidemics.
3. (*a*) Medical history of the more important campaigns, and the lessons to be learnt therefrom. (*b*) A knowledge of the Army Medical Service of the more important Powers. (*c*) The laws and customs of war so far as they relate to the sick and wounded.
4. A medical staff tour.

To assist candidates in the examination for promotion to the rank of major special facilities for study at the Royal Army Medical College are given and instruction in the special subjects.

In connection with this matter of promotion, there must be mentioned a very important concession, and that is that for distinguished service in the field, or for any other meritorious or distinguished service, including distinction in original investigation or research, a medical officer may be promoted to the next higher rank by brevet, which carries with it the pay and allowances of that rank.

Since the war began certain alterations have been made in this scheme of promotion. Some time ago it was announced that all officers admitted to the R.A.M.C. as lieutenants before the outbreak of war had been promoted to captain, irrespective of their length of service towards the usual qualifying period of 3½ years. Similarly last spring a long list of majors promoted to lieutenant-colonel was issued (over 170 all told), by which many officers obtained this promotion three or four or even five years before it would be due in the ordinary way. Many of these officers had not passed, many of them had had no opportunity of passing the examinations required as a condition of promotion in peace time. The number of promotions from captain to major has not much exceeded the ordinary.

### Retirement Regulations.

As regards retirement from the R.A.M.C., very definite rules are laid down. Thus, retirement may be compulsory from age, a surgeon-general having to go at sixty, a colonel at fifty-seven, and other officers at fifty-five, but it may also be voluntary, an officer being permitted to resign or retire at any time with the approval of the Army Council. The scale of retire-pay varies with rank and length of service. At present a director-general with three years' service in that appointment and thirty years' general service gets £1,125, a surgeon-general £730, a colonel with thirty years' service £547, a lieutenant-colonel £501, and a major after twenty years' service £375. Other officers with shorter periods of service and with lower rank receive gratuities on retiral. Thus, a major with six years' service in that rank gets £2,500, but with only three years' service £1,800, while an officer after five years' service in the rank of captain is entitled to £1,000. Of the officers thus voluntarily going into retirement, those with at least three, but not more than six, years' service may be allowed to join the reserve of officers for seven years, and they will receive while so situated a retaining fee of £25 a year.

Of the other conditions of service of an Army medical officer, it should be noted that he is entitled to sixty-one days of ordinary leave of absence on full pay during the year, and as regards sick leave, if granted on the recommendation of a Medical Board, he may draw full pay for a period not exceeding twelve months, which in special circumstances, such as loss of health due to tropical service or to active operations, may be extended, but it will not exceed eighteen months in all. Under certain conditions, too—wounds and injuries—pensions, as also gratuities, are granted to medical officers, and in the case of their dying, when either on the Active or Retired List, pensions may be granted to their widows and compassionate allowances to children.

The yearly rates for widows range from £40 to £120, and for children from £10 to £20 in ordinary peace service conditions. Higher rates are granted when the officer dies through war service or through injury sustained on duty. A gratuity may also be granted in addition to pension to the widow and children of an officer who has been killed in action or died of wounds received in action within seven years after having been wounded. The scale ranges from £100 to £1,100 for the widow and one-third of this amount for each child. In certain special circumstances annual allowances are made to the mother or sisters of an officer who has been killed in action or died of wounds received in action. The whole of the rules and regulations relating to these pensions and gratuities are laid down in the Army Pay Warrant.

[*See also page* 489.]

